# Haplotype frequencies in a sub-region of chromosome 19q13.3, related to risk and prognosis of cancer, differ dramatically between ethnic groups

**DOI:** 10.1186/1471-2350-10-20

**Published:** 2009-03-03

**Authors:** Mikkel H Schierup, Thomas Mailund, Heng Li, Jun Wang, Anne Tjønneland, Ulla Vogel, Lars Bolund, Bjørn A Nexø

**Affiliations:** 1 Bioinformatics Research Center, University of Aarhus, DK-8000 Aarhus C, Denmark; 2 Genetics and Ecology, Institute of Biology, University of Aarhus, DK-8000 Aarhus C, Denmark; 3 Institute of Human Genetics, University of Aarhus, DK-8000 Aarhus C, Denmark; 4 Beijing Genomics Institute at Shenzhen, Shenzhen, PR China; 5 Department of Biochemistry and Molecular Biology, University of Southern Denmark, DK-5000 Odense, Denmark; 6 Institute for Epidemiological Cancer research, The Danish Cancer Society, DK-2100 Copenhagen O, Denmark; 7 National Food Institute, Technical University of Denmark, DK-2860 Søborg, Denmark; 8 Institute for Science, Systems and Models, University of Roskilde, DK-4000 Roskilde, Denmark

## Abstract

**Background:**

A small region of about 70 kb on human chromosome 19q13.3 encompasses 4 genes of which 3, *ERCC1*, *ERCC2*, and *PPP1R13L *(aka *RAI*) are related to DNA repair and cell survival, and one, *CD3EAP*, aka *ASE1*, may be related to cell proliferation. The whole region seems related to the cellular response to external damaging agents and markers in it are associated with risk of several cancers.

**Methods:**

We downloaded the genotypes of all markers typed in the 19q13.3 region in the HapMap populations of European, Asian and African descent and inferred haplotypes. We combined the European HapMap individuals with a Danish breast cancer case-control data set and inferred the association between HapMap haplotypes and disease risk.

**Results:**

We found that the susceptibility haplotype in our European sample had increased from 2 to 50 percent very recently in the European population, and to almost the same extent in the Asian population. The cause of this increase is unknown. The maximal proportion of overall genetic variation due to differences between groups for Europeans versus Africans and Europeans versus Asians (the F_st _value) closely matched the putative location of the susceptibility variant as judged from haplotype-based association mapping.

**Conclusion:**

The combined observation that a common haplotype causing an increased risk of cancer in Europeans and a high differentiation between human populations is highly unusual and suggests a causal relationship with a recent increase in Europeans caused either by genetic drift overruling selection against the susceptibility variant or a positive selection for the same haplotype. The data does not allow us to distinguish between these two scenarios. The analysis suggests that the region is not involved in cancer risk in Africans and that the susceptibility variants may be more finely mapped in Asian populations.

## Background

**Figure 1:**
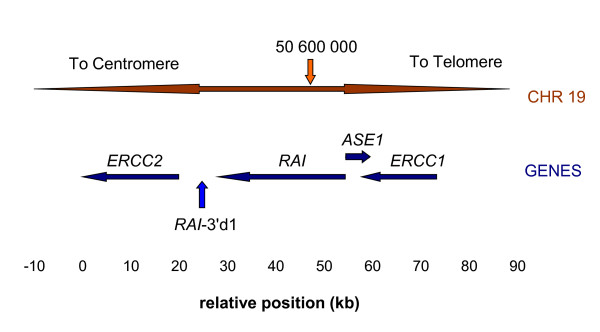
**Cartoon of the region investigated in the current study**. From [10].

A small region of about 70 kb on human chromosome 19q13.3 encompasses four genes of which three, *ERCC1*, *ERCC2*, and *PPP1R13L *(aka *RAI*) are related to DNA repair and cell survival, and one, *CD3EAP*, aka *ASE*1, may be related to cell proliferation. Evidence suggests that the genes are co-ordinately expressed [1] and the whole region may be related to the cellular response to external damaging agents [2] Figure 1 illustrates the region.

Studies of genetic epidemiology have implicated this region as a risk determinant for multiple types of cancer in Caucasians [3-8]. It may also be associated with cancer risk among Chinese [9]. Extensive searches in the region were carried out on the postmenopausal breast cancers of the prospective study "Diet, Cancer and Health" using nested, individually matched cases and controls. Each group comprised 434 persons, imbedded in the 29 549 women of the population based cohort. We have so far identified a polymorphism, called *RAI*-3'*d1*. The formal designation of the polymorphism is NT_011109.15g18147012dupATTTT(2_12). It is located 3' of the gene *PPP1R13L *and is the polymorphism with the strongest association to postmenopausal breast cancer among Danish women [10]. Recent data also point to a functional difference of the RAI-3'd1 alleles: Studies using electrophoretic mobility shift assays indicate that a typical "long" alleles having 9 repeats binds nuclear proteins with higher affinity than a typical "short" alleles (having 4 repeats). This suggests that the polymorphism has a role in transcription (K.K. Nissen et al, manuscript in preparation). However, *PPP1R13L *and part of *ERCC2 *are located in a haplotype-block with very limited recombination. Moreover, not all polymorphisms in the region have been tested, as some are located in regions of highly repetitive DNA, so that design of relevant assays is very difficult. Thus, a formal identification of the causative variation(s) is still lacking and it is possible that some of the untested polymorphisms are of significance. Certain other polymorphisms seem to play an additional role in specific cancers. For instance, a polymorphism in the 3' coding region of *ERCC2*, called *XPD*e23 or K751Q, may interact with the polymorphisms in or around *PPP1R13L *in determining risk of lung cancer [8]. Finally, the region, in particular the polymorphism *ASE1*e1 may also play a role in the prognosis of certain cancers such as multiple myeloma [11] and lung cancer (U. Vogel, et al, unpublished). Several polymorphisms in the ERCC1 region have been associated with survival among cancer patients [12-14].

Helgason et al. [15] reported that a gene associated with diabetes 2 susceptibility contained a variant haplotype that appeared to have been selected for in Asian and European populations. Thus, disease susceptibility variants may not always be selectively neutral even if they primarily manifest themselves after reproductive age (see also [16-19]). Indeed, the susceptibility variants in chromosome 19q13.3 appear to affect relatively young people and can therefore have a selection against them both directly through reproductive output and indirectly through caring for children and grandchildren after menopause. If this is the case, they are only expected to reach high frequency due to one of the following factors: 1) they have a positive effect on some other trait, 2) they are linked to a variant which is positively selected and thus are pulled to high frequency by genetic hitch hiking, 3) they have increased by genetic drift, e.g. during a population bottleneck.

We therefore surveyed the 19q13.3 region for differences in haplotype structure and frequencies in the HapMap populations of European, Asian and African descent. We found that the susceptibility haplotype has increased from 2 to 50 percent very recently in the European population, and to almost the same extent in the Asian population. The cause of this increase is unknown. The maximal F_st_-value closely matches the putative location of the susceptibility variant as judged from haplotype-based association mapping.

## Methods

### Ethical matters

This study was performed in compliance with the Helsinki declaration. The project was approved by the Science Ethical Committee of Copenhagen (j.nr: 01-345/93/(KF) 11-037/01/11-124/01). Written and verbal informed consent was obtained from all participants.

### HapMap populations

The DNA samples come from a total of 270 people. The Yoruba people of Ibadan, Nigeria, provided 30 trios (two parents and an adult child). In Japan, 45 unrelated individuals from the Tokyo area provided samples. In China, 45 unrelated individuals from Beijing provided samples. Thirty U.S. trios provided samples, which were collected in 1980 from U.S. residents with northern and western European ancestry by the Centre d'Etude du Polymorphisme Humain (CEPH). Data used was downloaded from the genome variation server (GVS) at University of Washington . This data was imported into Haploview 4.0 [20] where the haplotype block structure of 23 common markers in CEU was analysed in detail. Haplotype blocks in CEU were defined using the criterion of Gabriel et al. [21]: a block is created if 95% of informative (i.e. non-inconclusive) comparisons are "strong LD", i.e. with D' > 0.7 for > 95% of pairwise markers. The same blocks were manually defined in the YRI and Asian populations for comparison. All haplotypes with frequencies > 1% were displayed.

### Typing strategy

We sequenced the region spanning from the 5' portion of *ERCC2 *to the 3' portion of *ERCC1 *in 10 breast cancer cases and 10 controls. The actual number of reliable sequences varied somewhat from place to place. In total, we discovered 106 polymorphisms including those that were present in the NCBI's dbSNP. Of these 21 were found to contain very little variation and for 14 we could not develop a reliable assay due to repetitive sequences. Of the remaining 71 SNPs, we report results from complete typing of 58 In addition to these SNP we typed 4 more outside the region to span the full 70 kb region. A full list of the markers is available in the supplementary material from [10].

### Population differentiation (F_st_)

We downloaded the chromosome 19 genotype data for the populations from HapMap. For each shared SNP on the chromosome we calculated the F_st _statistic between the CEU population and the YRI population (with 32,310 shared SNPs) and between the CEU population and the pooled CHB and JPT populations (with 32,443 shared SNPs).

**Figure 2:**
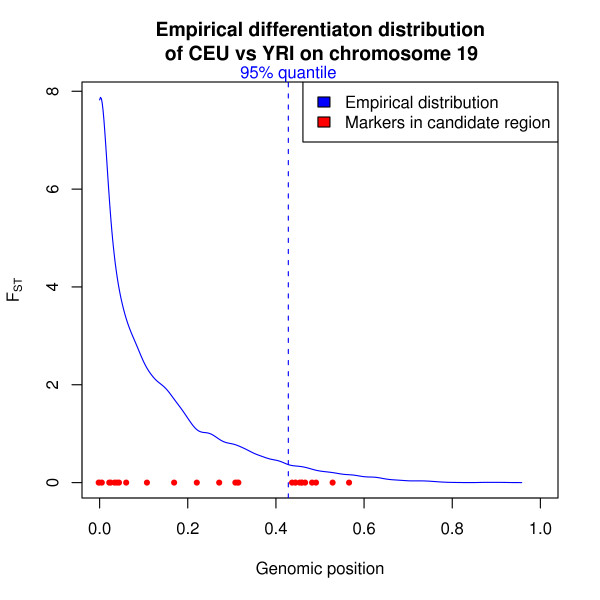
**Distribution of Fst values on chromosome 19 between Europe-Africa**. Vertical line shows the 95% percentile and the red dots the observed F_st _values in the candidate region.

To plot the distribution in Figure 2, we calculated the empirical density function using the *density *function in R. To obtain the 95% percentile we used the *quantile *function from R.

### Imputation of genotypes using FastPHASE

We pooled the case/control individuals with GVS individuals by considering the genotypes of markers that are not in the case/control individuals as missing data. We then imputed the most likely genotypes of the missing data using FastPHASE, and this way increased the number of markers in the case/control data from 58 SNPs to 125 SNPs. This allowed us to compare the inferred frequency of the key 11-marker haplotype block in the Breast cancer data set and test its association with disease as well as with previously identified associated markers, e.g. RAI-3'd1.

### Association mapping

We tested both typed and imputed SNPs for association with breast cancer. We tested each marker individually with a 3 × 2 χ^2^-test and tested haplotype-phenotype association using the Blossoc method [22].

**Figure 3:**
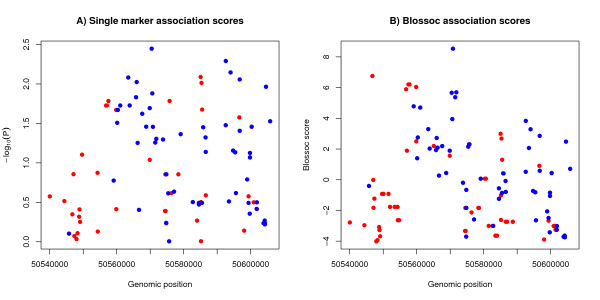
**Association analysis on typed and imputed markers**. A) Single marker association, B) Blossoc analysis. The blue dots indicate markers that were typed in the case/control data, while the red dots indicate markers that were imputed for the case/control individuals using GVS data.

For the single marker test we used the tool chisq_sma available from[23] and for the Blossoc analysis we used the Blossoc tool from [24] We ran chisq_sma with default options and Blossoc with options "-m5-fH". Of the 125 SNPs we could not compute the single marker statistics in 29 cases, due to too few counts in at least one of the cells of the contingency table. Figure 3A thus only shows 96 points, rather than 125. The Blossoc method can compute scores for all 125 SNPs.

## Results

In order to compare our case-control data to HapMap data [25] we first downloaded all SNP data on 19q13.3 from the European, Asian and Yoruban populations from the Human Genome Variation Server . We combined the European data from GVS with a Danish breast cancer case/control data set [10] and used FastPHASE [26] to impute the SNPs not typed in the case/control data but in the GVS data. Figure 3A shows the individual p-values of association to disease for typed SNPs (shown as blue dots) and imputed SNPs (shown as red dots). The association is highest (P = 3.58 × 10^-3^) at chromosome position 50,570,445, corresponding to the marker *RAI*-3'7. Figure 3B shows a haplotype based analysis, called Blossoc [22] of the association of SNPs with breast cancer. Blossoc uses hierarchical clustering of local haplotypes to estimate the position of the putative causative variant. The maximum association was located at 50,570,814, closely corresponding to the marker *RAI*-3'd3. With the imputed SNPs and haplotype analysis we conclude, as in the previous study, that the maximal association of SNPs with breast cancer in this region was located just 3' of PPP1R13L [10] and do not delimit the position of the causative genetic variant further.

**Figure 4:**
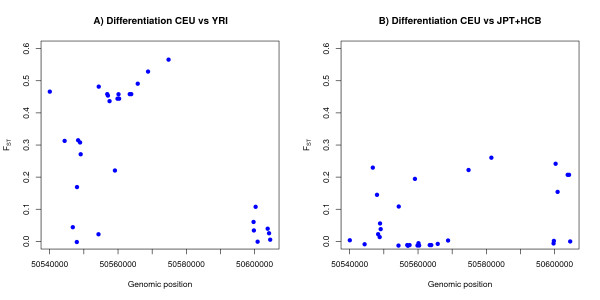
**F_st _values in the region: A) between Africa and Europe, B) between Europe and Asia**.

Next, we calculated F_st_, the proportion of overall genetic variation due to differences between groups for Europeans versus Africans (Figure 4A) and Europeans versus Asians (Figure 4B), using the HapMap SNPs. In the samples, Europeans and Africans share 29 SNPs in the region and Europeans and Asians share 30 SNPs in the region. A genetic difference between Europeans and Africans was distinctly present immediately 3' to the *PPP1L13L *and to the left, while limited differences were present to the right, with maximal values from chromosome position 50,568,000 to 50,575,000 (Figure 4A). A similar difference is not found when comparing Asia and Europe (see Figure 4B), but we observed a similar tendency in the comparison of Asians and Africans (not shown) albeit with slightly lower values and possibly shifted slightly to the left. Although we would generally consider an F_st_-value of 0.5 large, we still expect to find F_st _> 0.5 in numerous places in the genome. Figure 2 shows the empirical distribution of F_st _values between the HapMap European and African populations across the entire chromosome 19. The 95% percentile, shown as a vertical line, was 0.4281. The F_st _of the markers in the region are shown as red dots. Of the 29 SNPs in the region, 13 are among the 5% most differentiated SNPs on the chromosome, but there are many other SNPs with similarly high F_st _values. The relatively high differentiation combined with the known association with cancer, however, prompted our further investigation.

**Figure 5:**
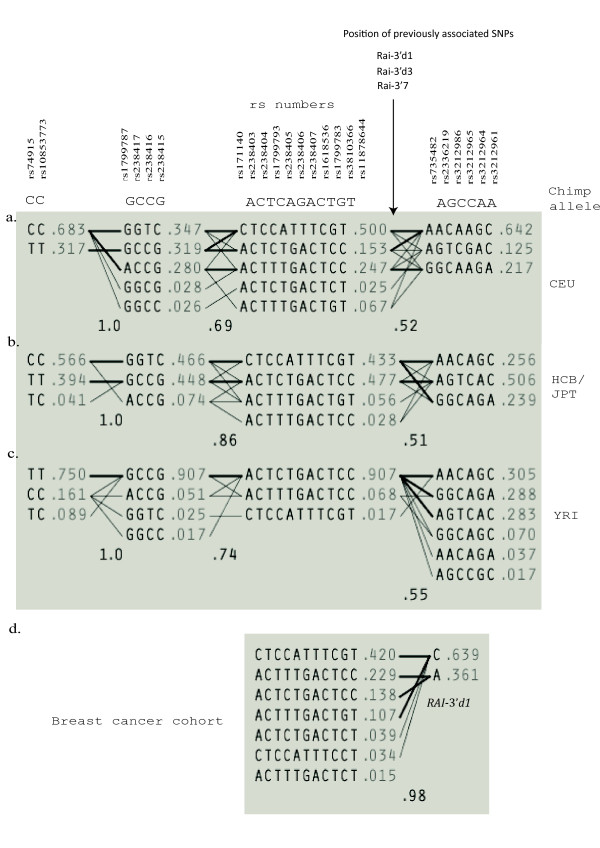
**The main haplotypes in the region for a) Europe), b) Asia and c) Africa**. The haplotypes blocks were defined applying the criterion of Gabriel et al. [21] on the CEU data. Haplotypes in different blocks are connected with thick lines if shared more than 10% and with thin lines if shared 1–10%. Numbers between blocks indicate average linkage disequilibrium measured as D' between adjacent blocks The ancestral state of each SNP was inferred from the chimpanzee sequence, using the genome variation server. The haplotype CTCCATTTCGT (block 3) has an increased frequency in CEU and consists mainly of derived alleles. It is an inferred risk variant in the Breast Cancer data set (panel d) and is in strong LD with previously identified susceptibility markers (position of these shown above Figure, and frequency of Rai3'd1 is shown in panel d).

To elucidate further the ethnic differences in this region of 19q13.3 we calculated the frequencies of haplotypes in the region for the three ethnic groups using the chimpanzee genome to identifying the ancestral form. The SNPs used for this analysis are shown in Figure 5. Haplotypes were phased by the default method in Haploview based on pedigree information from the trio making up the data sets for YRI and CEU. The haplotypes spanning the region of maximal F_st _could be broken down into several sub-regions with considerable recombination in between. A core region of 11 SNPs (from rs171140 to rs11878644 in Figure 5) seemed rather well preserved. In this core region one particular haplotype ACTCTGACTCC, which seemed closest related to the chimpanzee haplotype, constituted 91 percent of the African chromosomes, but only 15 percent of Europeans and 48 percent of the Asian. In contrast, the haplotype CTCCATTTCGT was only present on 2 percent of African chromosomes, but 50 percent European and 43 percent Asian chromosomes. Thus, major shifts of haplotype frequencies in this region seem to have taken place in the evolution of the European and Asian populations. Remarkably, the haplotype CTCCATTTCGT, which has increased in the European and Asian populations, is in very strong LD with the prime candidate SNP for association with breast cancer, RAI-3'd1 (Figure 5d), in that all chromosomes carrying the CTCCATTTCGT haplotype also carries the risk allele at RAI-3'd1. Therefore, the CTCCATTTCGT haplotype is also an inferred risk allele by a test of independence (P < 0.05).

## Discussion

In this paper, we have held together two different sets of DNA data, (1) a set of Danish women suffering from postmenopausal breast cancer, and (2) the HapMap DNA data from different ethnic groups. It appears that a haplotype in a region of high association with breast cancer has proliferated during the formation of the European population and to a somewhat lesser extent in the formation of the Asian population. In contrast, the most common allele among Africans, which also seem to be the ancestral form according to the Chimpanzee sequence, has diminished considerably in the "new" populations of Europe and Asia. It is therefore likely that the susceptibility haplotype is the derived state and that it increased in connection with the out-of-Africa expansion 60,000–100,000 years ago.

Remarkably, the accumulation of haplotypes runs contra to what we would expect, if risk of breast cancer were a selectable property. Specifically, the haplotype CTCCATTTCGT, which has accumulated about 30-fold in the transition from Africans to Europeans seems to be positively associated with cancer risk.

It is possible that other phenotypes, also associated with this haplotype, have exerted a stronger selective pressure in the opposite direction during the development of the derived populations, where adaptive evolution in response to changing environment appears to have been prominent but not yet disentangled [19,27,28]. Alternatively, the accumulation of the cancer-prone haplotype could be a result of genetic drift overriding weak selective forces in the periods, when the derived non-African populations were still quite small [16,29,30].

## Conclusion

We have analysed a region of 19q13.3 previously shown to be associated with cancer. We find that the polymorphisms shared between the CEU and YRI HapMap populations are highly divergent: 13 out of 29 SNPs in the region are in the 95% percentile of F_st _statistics for the chromosome.

A detailed analysis identified a haplotype found in low frequency (2%) in Africans but in high frequency in Europeans (49%) and Asians (43%). Based on a comparison with the Chimpanzee genome, we conclude that this haplotype is derived, and most likely has increased in frequency in the European and Asian populations since the out-of-Africa migration, rather than decreased in frequency in the African population.

The haplotype is associated with increased risk of cancer, including early postmenopausal breast cancer, and we would *a priori *expect selection to work against it. The increase in frequency in the European and Asian populations can be explained by either genetic drift or by selection for the haplotype. In the case of genetic drift, the reduced effective population size during the out-of-Africa migration could have lead to a reduction in selective pressure against the haplotype, allowing it to increase in frequency. Positive selection for the haplotype, in Europeans and Asians but not in Africans, could be caused either by the introduction of a new genetic variant on the haplotype background, or by the changes in environment factors in Europe and Asia. With the available data, we are not able to distinguish between these scenarios.

## Abbreviations

*ASE1*: Antisense *ERCC1*; CEU: CEPH Europeans from Utah; *ERCC1*: Excision-Repair, Complementing Defective, In Chinese Hamster, 1; *ERCC2*: Excision-Repair, Complementing Defective, In Chinese Hamster, 2; **F**_st_: proportion of overall genetic variation due to differences between ethnic groups; *PPP1R13L*: Protein Phosphatase 1, Regulatory Subunit 13-Like; *RAI*: RelA-Associated Inhibitor; *RAI*-3'd1: NT_011109.15 g.18147012ATTTT(2_11) coinciding with rs7255792; RAI-3'd3: rs3047560; *RAI*-3'7: rs7252567; YRI: Yorubans from Ibadan, Nigeria.

## Competing interests

The authors declare that they have no competing interests.

## Authors' contributions

AT collected the DNAs. BAN, UV performed the genotyping. MHS and TM performed the analyses. LH, WY participated in certain analyses. MHS, TM, BAN and LB planned the investigations and discussed the results. MHS, TM and BAN drafted the paper. All authors read and approved the final manuscript.

## Pre-publication history

The pre-publication history for this paper can be accessed here:



## References

[B1] Vogel U, Nexo BA, Tjonneland A, Wallin H, Hertel O, Raaschou-Nielsen O (2006). ERCC1, XPD and RAI mRNA levels in lymphocytes are not associated with lung cancer risk in a prospective study of Danes. Mutation research.

[B2] Laska MJ, Strandbygard D, Kjeldgaard A, Mains M, Corydon TJ, Memon AA, Sorensen BS, Vogel U, Jensen UB, Nexo BA (2007). Expression of the RAI gene is conducive to apoptosis: studies of induction and interference. Experimental cell research.

[B3] Dybdahl M, Vogel U, Frentz G, Wallin H, Nexo BA (1999). Polymorphisms in the DNA repair gene XPD: correlations with risk and age at onset of basal cell carcinoma. Cancer Epidemiol Biomarkers Prev.

[B4] Nexo BA, Vogel U, Olsen A, Ketelsen T, Bukowy Z, Thomsen BL, Wallin H, Overvad K, Tjonneland A (2003). A specific haplotype of single nucleotide polymorphisms on chromosome 19q13.2-3 encompassing the gene RAI is indicative of post-menopausal breast cancer before age 55. Carcinogenesis.

[B5] Rockenbauer E, Bendixen MH, Bukowy Z, Yin J, Jacobsen NR, Hedayati M, Vogel U, Grossman L, Bolund L, Nexo BA (2002). Association of chromosome 19q13.2-3 haplotypes with basal cell carcinoma: tentative delineation of an involved region using data for single nucleotide polymorphisms in two cohorts. Carcinogenesis.

[B6] Skjelbred CF, Saebo M, Nexo BA, Wallin H, Hansteen IL, Vogel U, Kure EH (2006). Effects of polymorphisms in ERCC1, ASE-1 and RAI on the risk of colorectal carcinomas and adenomas: a case control study. BMC cancer.

[B7] Vogel U, Hedayati M, Dybdahl M, Grossman L, Nexo BA (2001). Polymorphisms of the DNA repair gene XPD: correlations with risk of basal cell carcinoma revisited. Carcinogenesis.

[B8] Vogel U, Laros I, Jacobsen NR, Thomsen BL, Bak H, Olsen A, Bukowy Z, Wallin H, Overvad K, Tjonneland A (2004). Two regions in chromosome 19q13.2-3 are associated with risk of lung cancer. Mutation research.

[B9] Yin J, Vogel U, Ma Y, Qi R, Sun Z, Wang H (2007). A haplotype encompassing the variant allele of DNA repair gene polymorphism ERCC2/XPD Lys751Gln but not the variant allele of Asp312Asn is associated with risk of lung cancer in a northeastern Chinese population. Cancer genetics and cytogenetics.

[B10] Nexo BA, Vogel U, Olsen A, Nyegaard M, Bukowy Z, Rockenbauer E, Zhang X, Koca C, Mains M, Hansen B (2008). Linkage disequilibrium mapping of a breast cancer susceptibility locus near RAI/PPP1R13L/iASPP. BMC medical genetics.

[B11] Vangsted A, Gimsing P, Klausen TW, Nexo BA, Wallin H, Andersen P, Hokland P, Lillevang ST, Vogel U (2007). Polymorphisms in the genes ERCC2, XRCC3 and CD3EAP influence treatment outcome in multiple myeloma patients undergoing autologous bone marrow transplantation. Int J Cancer.

[B12] Moreno V, Gemignani F, Landi S, Gioia-Patricola L, Chabrier A, Blanco I, Gonzalez S, Guino E, Capella G, Canzian F (2006). Polymorphisms in genes of nucleotide and base excision repair: risk and prognosis of colorectal cancer. Clin Cancer Res.

[B13] Olaussen KA, Dunant A, Fouret P, Brambilla E, Andre F, Haddad V, Taranchon E, Filipits M, Pirker R, Popper HH (2006). DNA repair by ERCC1 in non-small-cell lung cancer and cisplatin-based adjuvant chemotherapy. The New England journal of medicine.

[B14] Park DJ, Zhang W, Stoehlmacher J, Tsao-Wei D, Groshen S, Gil J, Yun J, Sones E, Mallik N, Lenz HJ (2003). ERCC1 gene polymorphism as a predictor for clinical outcome in advanced colorectal cancer patients treated with platinum-based chemotherapy. Clin Adv Hematol Oncol.

[B15] Helgason A, Palsson S, Thorleifsson G, Grant SFA, Emilsson V, Gunnarsdottir S, Adeyemo A, Chen YX, Chen GJ, Reynisdottir I (2007). Refining the impact of TCF7L2 gene variants on type 2 diabetes and adaptive evolution. Nature Genetics.

[B16] Blekhman R, Man O, Herrmann L, Boyko AR, Indap A, Kosiol C, Bustamante CD, Teshima KM, Przeworskil M (2008). Natural selection on genes that underlie human disease susceptibility. Current Biology.

[B17] Gibson G (2007). Human evolution: Thrifty genes and the dairy queen. Current Biology.

[B18] Novembre J, Pritchard JK, Coop G (2007). Adaptive drool in the gene pool. Nature Genetics.

[B19] Sulem P, Gudbjartsson DF, Stacey SN, Helgason A, Rafnar T, Magnusson KP, Manolescu A, Karason A, Palsson A, Thorleifsson G (2007). Genetic determinants of hair, eye and skin pigmentation in Europeans. Nature Genetics.

[B20] Barrett JC, Fry B, Maller J, Daly MJ (2005). Haploview: analysis and visualization of LD and haplotype maps. Bioinformatics (Oxford, England).

[B21] Gabriel SB, Schaffner SF, Nguyen H, Moore JM, Roy J, Blumenstiel B, Higgins J, DeFelice M, Lochner A, Faggart M (2002). The structure of haplotype blocks in the human genome. Science.

[B22] Mailund T, Besenbacher S, Schierup MH (2006). Whole genome association mapping by incompatibilities and local perfect phylogenies. BMC Bioinformatics.

[B23] SMA. http://www.birc.au.dk/~mailund/sma.

[B24] Blossoc. http://www.birc.au.dk/~mailund/Blossoc.

[B25] Frazer KA, Ballinger DG, Cox DR, Hinds DA, Stuve LL, Gibbs RA, Belmont JW, Boudreau A, Hardenbol P, Leal SM (2007). A second generation human haplotype map of over 3.1 million SNPs. Nature.

[B26] Scheet P, Stephens M (2006). A fast and flexible statistical model for large-scale population genotype data: applications to inferring missing genotypes and haplotypic phase. Am J Hum Genet.

[B27] Balaresque PL, Ballereau SJ, Jobling MA (2007). Challenges in human genetic diversity: demographic history and adaptation. Human Molecular Genetics.

[B28] Bustamante CD, Fledel-Alon A, Williamson S, Nielsen R, Hubisz MT, Glanowski S, Tanenbaum DM, White TJ, Sninsky JJ, Hernandez RD (2005). Natural selection on protein-coding genes in the human genome. Nature.

[B29] Kryukov GV, Pennacchio LA, Sunyaev SR (2007). Most rare missense alleles are deleterious in humans: Implications for complex disease and association studies. American Journal of Human Genetics.

[B30] McCarthy MI, Abecasis GR, Cardon LR, Goldstein DB, Little J, Ioannidis JPA, Hirschhorn JN (2008). Genome-wide association studies for complex traits: consensus, uncertainty and challenges. Nature Reviews Genetics.

